# Validity and analysis of the Diabetes Injection Device Preference Questionnaire (DID-PQ)

**DOI:** 10.1186/s41687-020-00266-x

**Published:** 2020-12-09

**Authors:** Kristina S. Boye, Louis S. Matza, Brooke M. Currie, Karin S. Coyne

**Affiliations:** 1 Eli Lilly and Company, Indianapolis, IN USA; 2 Evidera, 7101 Wisconsin Avenue, Suite 1400, Bethesda, MD 20814 USA

**Keywords:** Preference, Injection devices, Type 2 diabetes, Patient-reported outcome measures, PRO, Dulaglutide, Semaglutide, Crossover study

## Abstract

**Introduction:**

The Diabetes Injection Device Preference Questionnaire (DID-PQ) was designed to assess patient preference between two non-insulin injection devices. In a recent crossover study, people with type 2 diabetes (T2D) completed the DID-PQ after performing mock injections with two non-insulin injection devices. The purpose of the current analysis was to use these data to assess construct validity of the DID-PQ and demonstrate one way to test whether there is a significant preference for one injection device over another.

**Methods:**

Data were from an open-label, multicenter, randomized, crossover study assessing preference between the dulaglutide and semaglutide injection pens. In addition to the 10-item DID-PQ, people with T2D completed a global item assessing overall preference. DID-PQ responses were compared to the global preference item (percent agreement, Gwet’s AC1, prevalence-adjusted and bias-adjusted Kappa [PABAK]). For each item of the DID-PQ, a two-sided binomial test assessed whether the difference in preference was statistically significant.

**Results:**

The sample included 310 participants (48.4% female; mean age = 60.0). The DID-PQ had minimal missing data. There was strong concordance (percent agreement > 78%) between the global preference item and all DID-PQ items except item 6, which assesses preference related to needle size (59.7%). The Gwet AC1 and PABAK statistics also indicated strong agreement between the global preference item and all DID-PQ items except item 6. There was a statistically significant difference (*p* < 0.0001) in preference on every DID-PQ item, with more participants preferring the dulaglutide device.

**Discussion:**

Patient preference has been recommended as a “major factor driving the choice of medication” in a consensus report by the American Diabetes Association and the European Association for the Study of Diabetes. Current findings suggest that the DID-PQ may be a useful tool for providing insight into preferences of people with T2D using non-insulin injectable medication.

## Introduction

Glucagon-like peptide-1 receptor agonists (GLP-1 RAs) are often recommended for treatment of type 2 diabetes (T2D) [1]. Medications in this class have demonstrated efficacy for glycemic control, along with a low risk of hypoglycemia and the potential benefit of weight loss [2–5]. The injectable GLP-1 RAs vary in terms of injection devices and treatment administration procedures, which could have an impact on patient preference.

Therefore, two patient-reported outcome (PRO) measures have been developed to assess patient perceptions of injection devices used to administer these non-insulin injectable medications: the Diabetes Injection Device Experience Questionnaire (DID-EQ) and the Diabetes Injection Device Preference Questionnaire (DID-PQ) [6]. The DID-EQ was designed to assess perceptions of a single injection device, and it has demonstrated reliability and validity in patients treated with GLP-1 RAs [7]. The DID-PQ was designed to assess preference between two non-insulin injection devices. This questionnaire has been used in two previous studies [7, 8]. In both studies, however, it was completed by a relatively small subset of patients who had used two non-insulin injection devices (*n* = 27 and *n* = 58). Therefore, it was not possible to draw conclusions about construct validity of the DID-PQ from these previous datasets.

In a recent crossover study with a larger sample, people with T2D performed mock injections with two non-insulin injection devices, and all participants completed the DID-PQ to report preferences between the devices [9]. Data from this study provide the first opportunity to examine performance of the DID-PQ in a larger sample. The purpose of the current analysis was to assess construct validity of the DID-PQ and demonstrate one way to test whether there is a significant preference for one injection device over another.

## Methods

### Study design

**Fig. 1:**
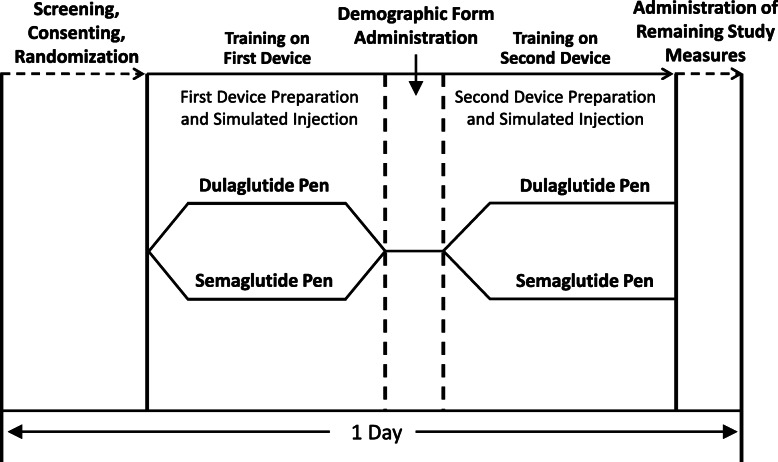
Crossover Study Design

Data were from an open-label, multicenter, randomized, crossover study (ClinicalTrials.gov identifier: NCT03724981) [9, 10] assessing patient preference for the dulaglutide single-use pen [11] and the semaglutide single-patient-use pen among injection-naïve patients with T2D [12]. The devices used in the study were those commercially available in the United States. The study design is illustrated in Fig. 1. Study participants were recruited at 13 clinical sites across the US, including nine general practice clinics and four endocrinology clinics. After providing consent to participate in the study, participants were randomly assigned to one of the two device orders (i.e., either dulaglutide or semaglutide first, followed by the other device). After being trained to use each device based on device instructions for use (IFU), participants performed all steps of injection preparation and administered mock injections into an injection pad. Further details of the study design, inclusion/exclusion criteria, and methods have been published previously [9].

### Measures

After completing training and performing mock injections with both devices, participants completed the measures described below. Both questionnaires were administered on paper forms and used the brand names (Trulicity for dulaglutide; Ozempic for semaglutide). The questionnaires included color images of the injection devices at the top of the page to avoid any confusion regarding which device corresponded to each question and response option.

#### Global preference item

The global preference item evaluated patient preference between the devices. The item asked “Overall, which device do you prefer?” Response options were Ozempic, Trulicity, or No Preference. All participants completed the global preference item before completing the DID-PQ.

#### Diabetes Injection Device Preference Questionnaire (DID-PQ)

The DID-PQ was designed to assess patient preferences between two non-insulin injection devices [6, 7]. The 10 questionnaire items were developed based on qualitative research with patients. Items 1 to 7 focus on preference related to specific characteristics of injection delivery systems. Items 8 to 10 are global items assessing preference based on overall satisfaction, ease of use, and convenience of the injection devices. Each item is rated on a five-point scale allowing respondents to indicate whether they prefer or strongly prefer one of the devices over the other. For each item, participants could also select the “no preference” response. As the five response options are categorical, mean scores are not calculated.

### Statistical analysis

Analyses were performed using data from participants who had (1) been randomized to a device order, (2) been exposed to both devices regardless of whether they successfully completed the mock injection, and (3) completed the global preference item. No imputations were performed for missing data. All statistical tests were two-sided with a significance level of 5%. Descriptive statistics (mean, standard deviation, range, and frequency) were used to summarize demographic and clinical characteristics, as well as responses to questionnaires.

The categorical response options of the DID-PQ cannot be treated as continuous scores. Therefore, correlations with a criterion measure that would typically be conducted to examine construct validity of PRO instruments cannot be used. Instead, the 10 DID-PQ items were compared to the global preference item using categorical analyses so that concordance between the two instruments could be assessed. For these analyses, the five DID-PQ response options were collapsed into three categories by combining the “prefer” and “strongly prefer” response options. Thus, the DID-PQ and global preference items had the same three levels of response: prefer dulaglutide device, prefer semaglutide device, and no preference between devices.

These three-level responses were compared to responses on the global preference item in three ways: (1) percent agreement, (2) Gwet’s AC1 statistic [13, 14], and (3) the prevalence-adjusted and bias-adjusted Kappa (PABAK) statistic [15]. The Gwet’s AC1 and PABAK statistics were used to assess concordance instead of the traditional Kappa statistic because Kappa is sensitive to uneven data distributions [16]. For example, when there is high agreement in situations with an uneven distribution of responses across the possible response options (e.g., high prevalence observed for one response option), Kappa may not accurately represent concordance [16]. Gwet’s AC1 is similar to Kappa, but it uses a different definition of chance agreement with a more realistic assumption that only a portion of the observed ratings will potentially lead to agreement by chance [13]. Thus, it is more robust to an uneven distribution of data. The PABAK statistic defines and incorporates both a bias index and prevalence index into its calculation of the estimate of chance agreement, therefore mitigating potential effects of rater bias and overall prevalence [15]. The Gwet AC1 and PABAK statistics were interpreted using benchmarks commonly used to interpret agreement statistics. For example, values over 0.80 are thought to indicate “almost perfect” agreement or “very good” agreement [17, 18].

To determine whether significantly more participants preferred one device over the other with regard to each item of the DID-PQ, comparisons between devices were performed according to the following steps: (1) participants who provided a neutral response for an item were dropped from analysis of that item; (2) for each item, responses were grouped into two categories (prefer dulaglutide device or prefer semaglutide device); and (3) a two-sided binomial test was performed to determine whether the difference in preference between the devices was statistically significant. This test assessed whether the proportion indicating preference for one of the two devices differed from 0.5. For each DID-PQ item, the null hypothesis was that the probability of preferring one of the devices was 0.5, which would indicate that an equal number of respondents preferred each device. If the binomial test yielded a significant *p*-value, then the null hypothesis could be rejected, which would mean that significantly more participants preferred one device over the other.

## Results

### Sample characteristics

A total of 310 participants were included in the sample, with half (*n* = 155) randomized to each group (i.e., either dulaglutide or semaglutide device first). Detailed demographic and clinical information has been previously published for this sample [9], and a selection of participant characteristics are presented Table 1.

**Table 1:** Demographic and Clinical Characteristics^a^

		Randomization Groups	
	Total Evaluable Sample ***N*** = 310	Dulaglutide Device First ***N*** = 155	Semaglutide Device First ***N*** = 155
**Age, years (mean, SD)**	60.0 (10.86)	60.5 (11.43)	59.5 (10.28)
Minimum-maximum	(30–86)	(34–86)	(30–83)
**Gender, female (n, %)**	150 (48.4%)	68 (43.9%)	82 (52.9%)
**Ethnicity: Hispanic or Latino (n, %)**^**b**^	39 (12.6%)	19 (12.3%)	20 (12.9%)
**Racial background (n, %)**			
Asian	10 (3.2%)	7 (4.5%)	3 (1.9%)
Black or African American	105 (33.9%)	52 (33.5%)	53 (34.2%)
White	155 (50.0%)	79 (51.0%)	76 (49.0%)
Other^c^	40 (12.9%)	17 (11.0%)	23 (14.8%)
**Employment status (n, %)**			
Full-time work	106 (34.2%)	57 (36.8%)	49 (31.6%)
Part-time work	43 (13.9%)	17 (11.0%)	26 (16.8%)
Retired	98 (31.6%)	55 (35.5%)	43 (27.7%)
Disabled	39 (12.6%)	18 (11.6%)	21 (13.5%)
Other^d^	24 (7.7%)	8 (5.2%)	16 (10.3%)
**Education level (n, %)**			
No college degree	201 (64.8%)	102 (65.8%)	99 (63.9%)
College degree	109 (35.2%)	53 (34.2%)	56 (36.1%)
**Type of clinical recruitment site**			
General practice	242 (78.1%)	120 (77.4%)	122 (78.7%)
Specialist	68 (21.9%)	35 (22.6%)	33 (21.3%)
**US geographic region**^**e**^			
Northeast	10 (3.2%)	6 (3.9%)	4 (2.6%)
Midwest	62 (20.0%)	29 (18.7%)	33 (21.3%)
South	203 (65.5%)	102 (65.8%)	101 (65.2%)
West	35 (11.3%)	18 (11.6%)	17 (11.0%)
**Duration of diabetes (mean years, SD)**	8.06 (6.76)	8.52 (7.03)	7.61 (6.47)
**Current oral medication to treat T2DM (n, %)**			
Sulfonylureas	74 (23.9%)	40 (25.8%)	34 (21.9%)
Biguanide	257 (82.9%)	130 (83.9%)	127 (81.9%)
DPP-4 inhibitors	20 (6.5%)	11 (7.1%)	9 (5.8%)
SGLT2 inhibitors	17 (5.5%)	10 (6.5%)	7 (4.5%)
Thiazolidinediones	7 (2.3%)	5 (3.2%)	2 (1.3%)
Combination pills	35 (11.3%)	15 (9.7%)	20 (12.9%)
**Most recent HbA1c value**			
Participants with HbA1c data (n, %)	304 (98.1%)	150 (48.4%)	154 (49.7%)
Mean (SD)	7.29 (1.42)	7.24 (1.35)	7.34 (1.48)

*Abbreviations*: *HbA1c* Hemoglobin A1C, *SD* Standard deviation, *DPP-4 inhibitors* Dipeptidyl peptidase 4 inhibitors, *SGLT2 inhibitors* Sodium-glucose co-transporter 2 inhibitors

^a^ Some of the information in this table was previously reported in the article presenting the clinical results of this study [9]

^b^ Of the 39 participants with ethnicity “Hispanic or Latino,” 14 were white, 25 were ‘other’ race

^c^ Race ‘other’ was self-reported as follows: American Indian or Alaska Native (*n* = 3); American Indian or Alaska Native + Black or African American + White (*n* = 2); American Indian or Alaska Native + White (*n* = 3); Asian + Black or African American (*n* = 1); American Indian or Alaska Native + Black or African American (*n* = 1); Native Hawaiian or Other Pacific Islander (*n* = 1); Hispanic or Hispanic American (*n* = 14); Indian (*n* = 1); Italian (*n* = 1); Latin (*n* = 1); Mediterranean (*n* = 1); Mexican (*n* = 5); Middle Eastern (*n* = 1); Puerto Rican (*n* = 1); ‘Mix with Caucasian/Indian’ (*n* = 1); Not specified (*n* = 3)

^d^ Employment ‘other’ was self-reported as follows: Homemaker/housewife (*n* = 9); Student (*n* = 1); Unemployed (*n* = 8); Stay-at-home parent (*n* = 4); Self-employed (*n* = 2)

^e^ Regions are based on the Census Bureau Regions listed here: http://www2.census.gov/geo/docs/maps-data/maps/reg_div.txt

### Validity of the DID-PQ

There were minimal missing data on the DID-PQ, as shown in Table 2. There was strong concordance (percent agreement > 78%) between the global preference item and nine of the 10 DID-PQ items (Table 2). Percent agreement was particularly high (> 91%) for the three DID-PQ global items assessing preference related to overall satisfaction, ease of use, and convenience (items 8, 9, and 10). The only DID-PQ item that did not have strong concordance with the global preference item was item 6, which asks about preference related to needle size (percent agreement = 59.7%). The Gwet AC1 and PABAK statistics were consistent with percent agreement, with results indicating strong agreement between the global preference item and all DID-PQ items except item 6 (Table 2).

**Table 2:** Agreement Between DID-PQ Items and the Global Preference Item Assessing Preferences between Two GLP-1 Receptor Agonist Injection Devices (*N* = 310)

	Patients whose DID-PQ Responses Matched Their Responses on the Global Preference Item							
DID-PQ Items^**a**^	Prefer Dulaglutide Device	No Preference between Devices	Prefer Semaglutide Device	Percent Agreement	Gwet AC_**1**_ (SE)	PABAK (SE)	Percent Disagreement	Missing DID-PQ Response
1. Ease of preparing device and medication for use	258	3	21	282 (91.0%)	0.90 (0.02)	0.86 (0.02)	28 (9.0%)	0 (0.0%)
2. Ease of fitting injection into your routine	233	9	24	266 (85.8%)	0.83 (0.03)	0.79 (0.03)	44 (14.2%)	0 (0.0%)
3. Ease of bringing device with you when you have to inject away from home	211	6	27	244 (78.7%)	0.74 (0.03)	0.68 (0.03)	66 (21.3%)	0 (0.0%)
4. Confidence that device provides the correct dose of medication every time	221	5	27	253 (81.6%)	0.78 (0.03)	0.72 (0.03)	57 (18.4%)	0 (0.0%)
5. Confidence that you are using device correctly	238	7	22	267 (86.1%)	0.84 (0.02)	0.80 (0.03)	42 (13.5%)	1 (0.3%)
6. Size of the needle	155	7	23	185 (59.7%)	0.47 (0.04)	0.40 (0.04)	124 (40.0%)	1 (0.3%)
7. Time it takes to prepare and inject medication	248	4	14	266 (85.8%)	0.84 (0.02)	0.79 (0.03)	43 (13.9%)	1 (0.3%)
8. Overall satisfaction with device	252	9	29	290 (93.5%)	0.92 (0.02)	0.90 (0.02)	20 (6.5%)	0 (0.0%)
9. Overall ease of using device (gated secondary outcome)	256	7	20	283 (91.3%)	0.90 (0.02)	0.87 (0.02)	27 (8.7%)	0 (0.0%)
10. Overall convenience of using device	257	6	25	288 (92.9%)	0.92 (0.02)	0.89 (0.02)	22 (7.1%)	0 (0.0%)

*Abbreviations*: *DID-PQ* Diabetes Injection Device Preference Questionnaire, *PABAK* Prevalence-adjusted and bias-adjusted kappa, *SE* Standard Error

^a^ The items are abbreviated in this table. The full item language is included in Table 3

### Significance testing of preferences between devices

For each item of the DID-PQ, a two-sided binomial test was performed to determine whether significantly more participants preferred one device over the other (Table 3). There was a statistically significant difference (*p* < 0.0001) in preference on every item of the DID-PQ with significantly more participants reporting a preference for the dulaglutide injection device.

**Table 3:** Significance Testing for Difference in Preference between Devices on Each Item of the DID-PQ (*N* = 310)

DID-PQ Items	N^**a**^	Prefer Dulaglutide Device^**b**^ n (%)	Prefer Semaglutide Device^**c**^ n (%)	***p***-value^**d**^
1. Ease of preparing the injection device and medication for use	302	280 (92.7%)	22 (7.3%)	<.0001
2. Ease of fitting the injection into your routine	260	235 (90.4%)	25 (9.6%)	<.0001
3. Ease of bringing the injection device with you when it is necessary to inject away from home	260	219 (84.2%)	41 (15.8%)	<.0001
4. Confidence that the injection device provides the correct dose of medication every time	270	234 (86.7%)	36 (13.3%)	<.0001
5. Confidence that you are using the injection device correctly	269	245 (91.1%)	24 (8.9%)	<.0001
6. The size of the needle	188	159 (84.6%)	29 (15.4%)	<.0001
7. The time it takes to prepare and inject each dose of medication	285	270 (94.7%)	15 (5.3%)	<.0001
8. Overall satisfaction with the injection device	285	255 (89.5%)	30 (10.5%)	<.0001
9. Overall ease of using the injection device (gated secondary outcome)	290	269 (92.8%)	21 (7.2%)	<.0001
10. Overall convenience of using the injection device	293	267 (91.1%)	26 (8.9%)	<.0001

*Abbreviations*: *DID-PQ* Diabetes Injection Device Preference Questionnaire

^a^ Excludes all neutral and missing responses to DID-PQ (total possible *N* = 310)

^b^ Includes DID-PQ responses of “strongly prefer dulaglutide” and “prefer dulaglutide”

^c^ Includes DID-PQ responses of “strongly prefer semaglutide” and “prefer semaglutide”

^d^ The *p*-values are from a two-sided binomial test for each DID-PQ item to determine whether significantly more participants preferred one device over the other. This test assessed whether the proportion of patients indicating preference for one of the two devices differed from 0.5. A significant *p*-value means that significantly more participants preferred one device over the other. Patients with no preference between devices were excluded from this analysis

## Discussion

Patient preference has been recommended as a “major factor driving the choice of medication” in a consensus report by the American Diabetes Association and the European Association for the Study of Diabetes [1]. To collect and interpret patient preference data, well-designed and valid measurement tools are needed. Current findings suggest that the DID-PQ may be a useful tool for providing insight into preferences of people with T2D using GLP-1 receptor agonists. While a single global item can be used to assess injection device preference, the DID-PQ can provide a more detailed assessment of factors contributing to this preference, including ease of use, convenience, overall satisfaction, and details of the injection experience.

Concordance with the global preference item supports the construct validity of the DID-PQ. Item 6 of the DID-PQ, which assesses preference related to needle size, had the lowest concordance (59.7% agreement). Although needle size is an important factor for some patients [6], this item may not have yielded consistent data because participants were injecting into an injection pad rather than injecting themselves. Therefore, they did not personally experience the feeling of injecting with either needle, and the factors that participants considered when responding to this question are unclear and may have varied widely. Future research involving actual injections rather than mock injections may be necessary to assess validity of DID-PQ item 6.

In addition to examining validity, the study provides a parsimonious and easily interpretable method for examining whether preference for one device over another is statistically significant (Table 3). This analysis approach excludes neutral (i.e., no preference) responses. For situations when it may be important to consider the number of neutral responses (which were relatively rare in the current study; Table 3), the Prescott test can be used to determine whether there was a statistically significant difference in preference while accounting for the frequency of respondents with no preference [19, 20]. The Prescott test was used in the original analysis of data from the current study, with similar statistically significant results favoring the dulaglutide device on all 10 items of the DID-PQ [9].

The structure of the DID-PQ does not allow for typical psychometric analyses, and the resulting limitations need to be considered. Unlike a PRO measure of symptoms or health-related quality of life, the items of the DID-PQ do not have ordinal response options ranging from lowest to highest on a particular construct, and item scores cannot be aggregated into subscales for analysis of continuous data. Instead, DID-PQ items yield categorical data representing preference. Therefore, it is not possible to assess internal consistency reliability with Cronbach’s alpha, test-retest reliability with intra-class correlations, or convergent validity with Spearman correlations. Furthermore, there are not generic instruments or validated gold standard criterion measures that may be used for assessment of construct validity. While the current categorical analyses support construct validity of the DID-PQ via comparisons to a single item assessing global preference, it is not possible to thoroughly investigate reliability or validity of the instrument using common psychometric methods. Future research with the DID-PQ may provide further confidence in its validity.

There may also be limitations associated with the mock injection procedures. Participants were trained on each device prior to injecting each pen into an injection pad. Participants did not inject themselves with medication. Some aspects of the injection experience, such as comfort related to needle size or liquid volume, were not apparent during these procedures. It is possible that some DID-PQ responses could have been different if participants had injected themselves instead of the injection pad. Still, participants were thoroughly trained on both injection devices, and they performed all parts of the injection process. Therefore, their DID-PQ responses were likely based on a good understanding of both devices.

Despite these limitations, the DID-PQ represents a step forward for assessment of patient preference between injection devices. For preference to inform clinical decisions, measurement tools focusing on comparisons between treatments will be necessary. Since the DID-PQ has been useful in several studies, perhaps it could be a model for development of questionnaires designed to assess preference among other treatments across a range of medical conditions.

## Data Availability

The study presented in this manuscript has been posted to ClinicalTrials.gov (NCT03724981), and data are available upon request.

## Abbreviations

DID-EQ: Diabetes Injection Device Experience Questionnaire
DID-PQ: Diabetes Injection Device Preference Questionnaire
GLP-1: Glucagon-like peptide-1
IFU: Instructions for use
PABAK: Prevalence-adjusted and bias-adjusted Kappa
PRO: Patient-reported outcome
RA: Receptor agonist
T2D: Type 2 diabetes
